# Improving Psychiatric Input for Older People with Dementia and Delirium in the Acute Hospital

**DOI:** 10.1192/bjo.2026.11364

**Published:** 2026-06-30

**Authors:** Thomas Coton, Charlotte Forbes, Catherine Gothard, Merryn Anderson

**Affiliations:** 1 Royal Devon University Healthcare NHS Foundation Trust, Exeter, United Kingdom; 2 Devon Partnership NHS Trust, Exeter, United Kingdom

## Abstract

**Aims:**

Older People’s Mental Health (OPMH) patients admitted to acute hospitals often experience fragmented care, particularly those with dementia or delirium. Delirium is associated with increased mortality and morbidity and prolonged hospital stays; yet it remains under-recognised and sub-optimally managed. National guidance emphasises earlyidentification, prompt access to mental health expertise, and collaborative multidisciplinary management. Locally, staff reported delays in psychiatric review, uncertainty in delirium management, and inconsistent use of cognitive screening tools.

This Quality Improvement (QI) Project aimed to:

1. Improve timely psychiatric input for OPMH patients admitted to the acute hospital.

2. Increase recognition and documentation of delirium, and behavioural/psychological symptoms of dementia (BPSD).

3. Enhance staff confidence in managing delirium/BPSD through education and standardised pathways.

**Methods:**

Using QI methodology, we conducted three Plan–Do–Study–Act(PDSA) cycles over a twelve-month period.

Interventions included streamlining patient allocation processes through enhanced morning triage meetings; contributing to dementia strategy group initiatives; establishing weekly liaison meetings between psychiatry and dementia/delirium nursing teams; developing liaison ‘champion’ roles; and delivering a programme of internal and external teaching.

Quantitative data was collected monthly from mental health and acute trust electronic patient records and qualitative data by anonymous questionnaires.

**Results:**

Following implementation:

Response time within 24 hours showed special-cause improvement (78.5% at baseline to 82.7% post-intervention); however, the process is still not stable enough to reliably meet the 90% target.

There was no sustained improvement in use of cognitive screening tools although there was sustained improvement in documentation of delirium diagnostic features (attention, fluctuation, cognitive change; 21% to 74%).

There was a trend to improvement in deprescribing and reduction in security team intervention. There was a sustained reduction in need for Mental Health Act referral (16.4% at baseline to 6.9% post-intervention) and reduction in average length of stay (33 days at baseline to 25 days post-intervention). There was a trend of increasing use of acute sedatives, but this change did not reach significance.

**Conclusion:**

Qualitative feedback highlighted improved communication, increased confidence, clearer escalation processes, and reduced distress among patients with BPSD.

Simple, low-cost interventions–education, standardised pathways, and collaborative daily reviews–enhanced both the timeliness and quality of psychiatric input. Future work includes sustaining improvements across wards, improving completion of standardised screening tools and evaluating patient-centred outcomes such as length of stay and readmission. Planned next interventions include development of non-medical prescribing protocols and deeper service change in the trust in collaboration with the older people’s mental health teams.

